# Migraine and Hormonal Contraception in Gynecological Outpatient Care—Cross-Sectional Study among Practicing Gynecologists in Germany

**DOI:** 10.3390/jcm12041434

**Published:** 2023-02-10

**Authors:** Mira P. Fitzek, Elisabeth Storch, Lucas H. Overeem, Pia Kull, Maria Terhart, Kristin S. Lange, Uwe Reuter, Bianca Raffaelli

**Affiliations:** 1 Department of Neurology, Charité—Universitätsmedizin Berlin, 10117 Berlin, Germany; 2 Universitätsmedizin Greifswald, 17489 Greifswald, Germany; 3 Clinician Scientist Program, Berlin Institute of Health at Charité (BIH), 10178 Berlin, Germany

**Keywords:** migraine, migraine aura, contraception, women’s health

## Abstract

Hormonal contraception (HC) can influence the migraine burden and should be considered in the comprehensive management of women with migraine. In this study, we aim to investigate the influence of migraine and migraine aura on the prescribing behavior of combined oral contraception (COC) and progestogen monotherapy (PM) in gynecological outpatient care. From October 2021 to March 2022, we performed an observational, cross-sectional study using a self-administered online-based survey. The questionnaire was distributed by mail and e-mail among 11,834 practicing gynecologists in Germany using the publicly available contact information. A total of 851 gynecologists responded to the questionnaire, of whom 12% never prescribe COC in the presence of migraine. Further 75% prescribe COC depending on the presence of limiting factors such as cardiovascular risk factors and comorbidities. When deciding to start PM, migraine appears to be less relevant, as 82% prescribe PM without restrictions. In the presence of aura, 90% of gynecologists do not prescribe COC at all, while PM is prescribed in 53% without restrictions. Almost all gynecologists reported to be actively involved in migraine therapy by having already initiated (80%), discontinued (96%), or changed (99%) HC due to migraine. Our results reveal that participating gynecologists actively consider migraine and migraine aura before and while prescribing HC. Gynecologists appear cautious in prescribing HC in patients with migraine aura.

## 1. Introduction

Migraine is a frequent neurological disease with a prevalence peak in women of childbearing age [1,2]. It ranks second among causes of disability worldwide and is the leading cause of disability in young women [3,4]. In the western population, the majority of women aged 15–49 use contraception, with hormonal contraception (HC) being one of the most commonly used methods [5].

Estrogen-containing contraception [6,7] and migraine, especially migraine with aura [8,9,10,11,12], are both independently associated with an increased risk of ischemic stroke. Therefore, the safety of HC in women with migraine remains controversial, and gynecologists are often faced with the challenge of selecting appropriate contraception. The risk of ischemic stroke with the use of estrogen depends on the dose, with high-dose formulations having the highest risk [13]. Ultra-low-dose formulations, containing less than 20 μg of ethinyl estradiol, do not pose an increased risk of stroke in healthy nonsmokers [6]. Progestogen monotherapy (PM) carries no additional risk of cardiovascular events [14].

Apart from the associated cardiovascular risks, the use of HC can also have preventive potential in migraine, especially in patients with a perimenstrual increase in migraine frequency and severity. Selected preparations can lead to a reduction of the migraine burden by eliminating the physiological fluctuations in sex hormones [15,16]. Treatment schemes that aim to keep estrogen concentrations stable, such as combined hormonal contraception (COC) used in continuous or extended cycles (shortened hormone free interval) or PM, can reduce menstrual-related migraine by up to 80% [17].

The European Headache Federation (EHF) and European Society of Contraception and Reproductive Health (ESC) consensus statement from 2017 recommends the use of PM or non-hormonal contraception for migraine with aura [11]. In migraine without aura, low-dose estrogen-containing contraception may be used in the absence of additional risk factors [6,11]. In clinical practice, HC is predominantly prescribed by gynecologists, while the prescription of migraine prophylaxis mainly belongs to the field of neurology. In this study, we aimed to investigate if the presence of migraine and migraine aura has an impact on the prescription of HC among German gynecologists and examined potential factors influencing the decision-making process.

## 2. Materials and Methods

### 2.1. Study Design, Setting and Procedure

This was an observational, cross-sectional study among practicing gynecologists in Germany. Data were collected between October 2021 and March 2022 using a standardized online questionnaire. The questionnaire was distributed as a link generated through RED Cap application (REDCap 12.0.33-© 2022 Vanderbilt University, Nashville, TN, USA) via e-mail and letter among all practicing gynecologists in non-university outpatient clinics in Germany (*n* = 11,881). In order to strengthen the response rate, we additionally sent out three reminder e-mails after the first invitation to participate. Contact information was publicly available through the corresponding Association of Statutory Health Insurance (“Kassenärztliche Vereinigung”).

### 2.2. Survey

The survey was a dynamic branching questionnaire that was purpose-built designed by the authors, containing up to 29 questions. The questionnaire was not validated. It was divided into five subunits, which are designed to collect information on the following topics: 1. Demographics; 2. Prescription of COC in the presence of migraine; 3. Prescription of PM in the presence of migraine; 4. Modification (initiation, discontinuation, change) of hormonal contraceptive treatment due to migraine; and 5. Management of patients suffering from migraine (e.g., referral to primary care physicians/neurologists) (see Supplementary Material File S1 for full survey). In questions about migraine, we first asked about migraine in general without sub-specification (hereafter referred to as migraine) and then specifically about migraine with aura. In some questions about the prescription of COCs, we distinguish between a classic cycle (21 days of hormone use +7 days of hormone-free interval) and an extended cycle (shortened or no hormone-free interval). Force-response validation was included in every section. Questions on the primary endpoint (prescription of hormonal contraction in migraine/migraine aura) were designed to be mandatory to complete before proceeding to the next section to minimize missing values. Information on descriptive characteristics and secondary endpoints such as modification of hormonal treatment due to migraine or further treatment of migraine patients was voluntary. Accordingly, number of participants for different questions may vary.

### 2.3. Statistical Analysis

For statistical analysis, we used IBM SPSS Statistics (IBM SPSS Statistics ©; 23.0, for Mac). For questions with the option not to answer, we classified non-answering as missing values. The total number of respondents per question (*n*) is indicated in parentheses in each case. We conducted descriptive analyses for sociodemographic characteristics and questions about prescribing behavior of hormonal contraceptives. Categorial variables are presented as absolute numbers (*n*) and relative frequencies (%), whereas means, min.-max., and standard deviations are used for continuous variables. To examine differences in the frequency of obtaining migraine anamnesis before starting therapy with COC vs. PM, we performed a chi-square test. In cases with the option to give multiple answers, relative frequencies may exceed 100%. For the comparison of age between subgroups, we used the Mann–Whitney U test, due to the non-normal distribution of data (assessed with the Shapiro–Wilk test). Figures were generated using PRISM software for Mac. (GraphPad Prism ©; version 8.4.3 (471), for Mac, GraphPad Software, Boston, MA, USA).

## 3. Results

**Figure 1:**
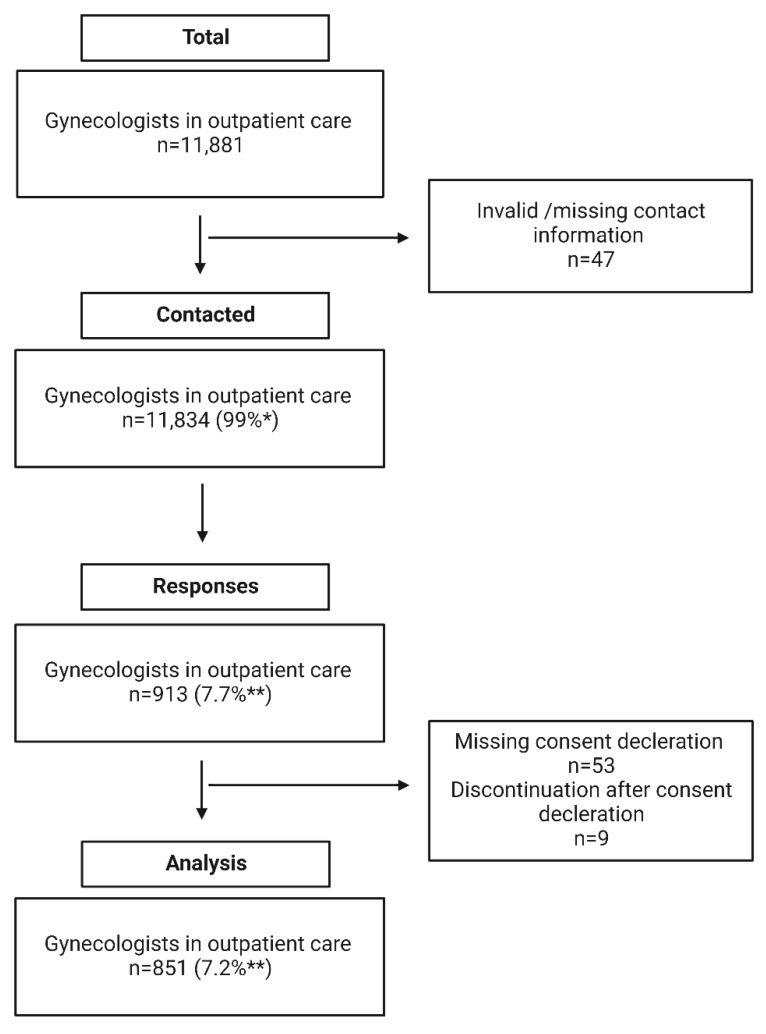
Recruitment; * percent of total population, ** percent of contacted gynecologists.

Out of *n* = 11,834 contacted gynecologists, *n* = 913 answered the questionnaire over the 6 months (October 2021–March 2022) in which the survey was available. Due to missing informed consent, *n* = 53 participants had to be excluded. Another nine participants did not answer a single question after giving informed consent and were, therefore, also excluded from the analysis. Accordingly, we analyzed the questionnaires of a total of *n* = 851 participants (Figure 1), of which *n* = 841 completed the questionnaire. This corresponds to a response rate of 7.2% for the entirety of practicing gynecologists in Germany. Response rates varied considerably depending on the federal state, with 20% for Thuringia and only 5% for Hesse.

### 3.1. Demographics

Table 1 shows the key demographic characteristics of study participants. The average age of respondents was 52 ± 8 (range: 33–78) years. The majority were women (79%; *n* = 669/845), with a clinical focus in general gynecology (96%; *n* = 811/842) and a work experience of over 10 years (64%; *n* = 542/847). Most participants (70%; *n* = 594/844) reported to have 50 to 150 patient visits weekly. Participating gynecological clinics were located in both low-population regions and large cities and were fairly evenly distributed in terms of population density: rural (20%; *n* = 170/846); small town (<100,000 inhabitants; 37%; *n* = 317/846); large city (100,000–500,000 inhabitants; 22%; *n* = 185/846); and large city (>500,000 inhabitants; 21%; *n* = 174/846).

### 3.2. Hormonal Contraception and Migraine in General

**Figure 2:**
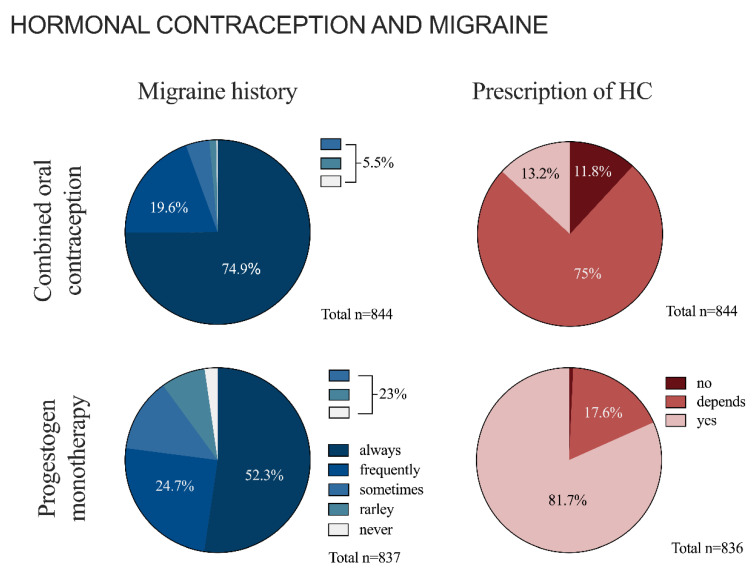
Hormonal contraception and migraine. The figure shows the absolute frequencies of asking about migraine (including migraine with and without aura) before initiating treatment with COC or PM, as well as prescribing patterns of COC and PM in the case of concomitant migraine.

Participating gynecologists reported to ask regularly about migraine prior to beginning any HC (Figure 2). A migraine history is obtained significantly more often before COC than PM treatment (χ^2^(1) = 103.97, *p* < 0.001, φ = 0.25/χ^2^(1) = 47, *p* < 0.001, φ = 0.21). Gynecologists who regularly (always or frequently) asked about migraine before starting a COC (*n* = 727/767) tend to be younger than gynecologists who reported to do so only rarely or sometimes (*n* = 40/767) (52 ± 0.3 age in years [SEM] vs. 55 ± 1.5 age in years [SEM], respectively; U = 12,090, *z* = −1.797, *p* = 0.072, Mann–Whitney-U). Regarding the frequency of obtaining a migraine history prior to prescribing a PM, no age-related differences could be observed (U = 43,387, *z* = −1.275, *p* = 0.202, Mann–Whitney-U).

The presence of migraine influences gynecologists in their decision whether to prescribe COC (Figure 2): 12% (*n* = 100/844) of participants never prescribed COC for women suffering from migraine. Another 75% (*n* = 633/844) would do so only to a limited extent. The proportion of gynecologists who prescribed COC without restrictions in patients suffering from migraine (*n* = 104/190) was significantly younger than those who never prescribed a COC in migraine patients (*n* = 86/190) (50 ± 0.8 age in years [SEM] vs. 54 ± 0.9 age in years [SEM], respectively; U = 3115.5, *z* = −3.598, *p* < 0.001, Mann–Whitney-U). When asked about potential factors influencing their decision (multiple answers allowed), the presence of cardiovascular risk factors (not further specified) (78%, *n* = 494/633) and other comorbidities (71%, *n* = 447/633) were listed most frequently (Table 2).

The presence of migraine appears to be less relevant when deciding to start HC with PM: Most participants (82%; *n* = 683/836) prescribed PM regardless of a potential migraine diagnosis (Figure 2). The remaining 18% (*n* = 147/836) prescribed PM only conditionally, naming cardiovascular risk factors (66%, *n* = 97/147) as the prime influencing factors (Table 2).

### 3.3. Hormonal Contraception and Migraine with Aura

**Figure 3:**
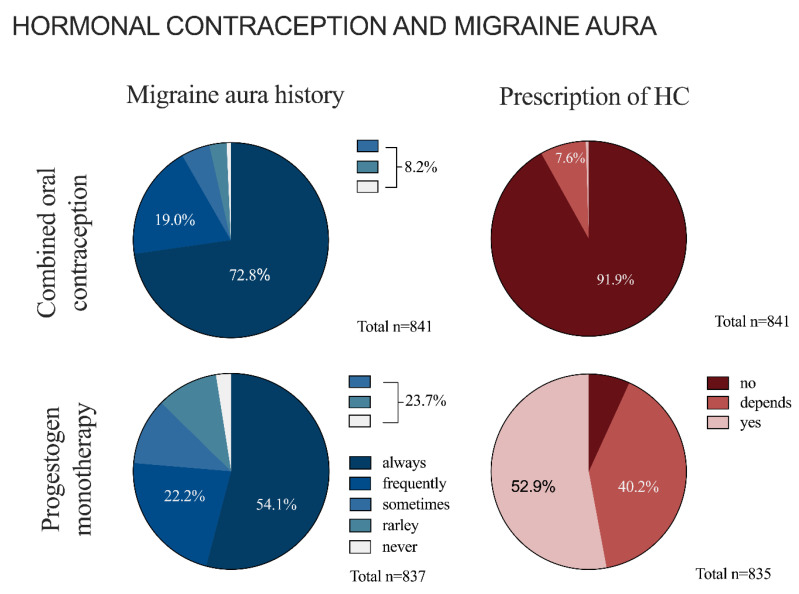
Hormonal contraception and migraine aura. The figure shows the absolute frequencies of asking about migraine aura before initiating treatment with COC or PM, as well as prescribing patterns of COC and PM in the case of concomitant migraine aura.

The analyzed cohort of gynecologists often asks specifically about migraine aura before prescribing HC (Figure 3). Questions about migraine aura are asked more frequently prior to COC than PM treatment (χ^2^(1) = 47, *p* < 0.001, φ = 0.21). Gynecologists who regularly (always or frequently) asked for migraine aura prior to prescribing COC (*n* = 708/763) are significantly younger than gynecologists who asked only rarely or sometimes (*n* = 55/763) (52 ± 0.3 age in years [SEM] vs. 56 ± 1.2 age in years [SEM], respectively; U = 14,076, *z* = −3.428, *p* = 0.001, Mann–Whitney-U). Regarding the frequency of obtaining a migraine aura history prior prescribing a PM, no age-related differences could be observed (U = 44,964, *z* = −0.523, *p* = 0.6, Mann–Whitney-U).

The majority of gynecologists (92%, *n* = 773/841) never prescribed COC for women with migraine with aura. Another 8% (*n* = 64/841) only prescribed COC under certain conditions (Table 3).

Regarding PM, more than half of the participating gynecologists (*n* = 442/835; 53%) prescribed this kind of HC without reservations in migraine with aura. Another 40% (*n* = 336/835) would do so only after taking certain factors into account (Table 3). A proportion of 7% (*n* = 57/332) never prescribed PM in migraine with aura. Gynecologists who prescribed PM in migraine aura without restriction (*n* = 408/453) appear to be significantly younger than those who would never prescribe PM for migraine with aura (*n* = 45/453) (51 ± 0.4 age in years [SEM] vs. 54 ± 1.4 age in years [SEM], respectively; U = 6840.5, *z* = −2.809, *p* = 0.005, Mann–Whitney-U).

### 3.4. Modification of Hormonal Treatment due to Migraine

**Figure 4:**
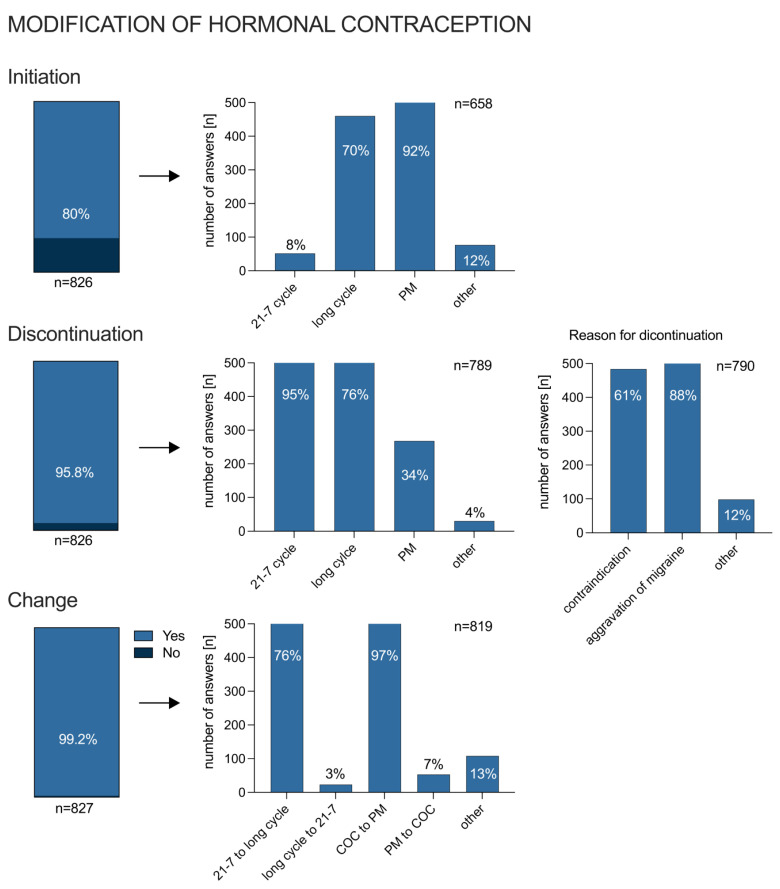
Modification of hormonal contraception due to migraine categorized into initiation, discontinuation and change, as well as reasons in the case of discontinuation of hormonal therapy.

Almost all participating gynecologists initiated (80%; *n* = 661/826), discontinued (96%; *n* = 791/826), and/or changed (99%; *n* = 820/827) a HC due to migraine. Figure 4 shows the type of modification in hormonal therapy and the respective frequencies. When asked how gynecologists would proceed with patients with migraine (multiple choices), the majority said they would refer them to a neurologist (92%, *n* = 736/826), followed by a referral to a primary care physician (28%, *n* = 229/826). Only 10% (*n* = 83/826) of respondents said they would treat migraine patients regarding the migraine themselves.

## 4. Discussion

In this observational study, we analyzed the influence of migraine and migraine aura on the prescription of hormonal contraception in gynecologist outpatient care. Our results reveal that gynecologists actively consider the presence of migraine in general and migraine with aura before prescribing hormonal contraception (HC). In women with migraine, gynecologists tend to prescribe progesterone monotherapy (PM) more often than combined oral contraception (COC), which is in line with currently valid guidelines [11]. This cohort of gynecologists, and especially the older participants, seems to be somewhat cautious in prescribing HC in patients with migraine, especially with aura, because of existing limiting factors like cardiovascular risk factors.

Without HC, the absolute risk of an ischemic stroke in women aged 20 to 44 with migraine without and with aura amounts to 4/100,000 and 5.9/100,000, respectively, and increases to 10/100,000 and 14.5/100,000 under the use of HC [11]. The risk of a stroke increases only with estrogen-containing contraception and remains unchanged with estrogen-free compounds [6,7,8,11,18]. In line with this, participating gynecologists are very reluctant in prescribing COC for migraine with aura and are more likely to resort to contraception without estrogen using PM. However, concerns about cardiovascular events appear to cause a significant proportion of gynecologists to hesitate even in prescribing PM. After all, 40% of participants prescribed PM in migraine with aura only with reservations, naming cardiovascular risk factors to be influencing factors, even though there are no contraindications to the use of PM in women with migraine and aura, as it is not associated with any additional stroke risk [14]. In fact, current guidelines recommend PM in migraine with aura when HC is desired. A restrictive prescription may unnecessarily impede access to HC for young women with migraine with aura. Interestingly, gynecologists’ prescribing behavior differed between age groups in two specific scenarios: prescription of COC in migraine and of PM in migraine aura. In detail, gynecologists who reported to prescribe COC in migraine without restrictions were significantly younger than those who reported to never prescribe COC in migraine. The same was observed for the prescription of PM in migraine aura. At least in the context of COC, one could speculate that this reflects changes in treatment recommendations over recent years that have softened the previous recommended strong advice against the use of COC for any patients suffering from migraine.

The use of hormonal contraception can influence the burden of migraine in both ways. Among women with migraine using HC, 18–50% notice a worsening of migraine, 3–35% report an improvement, and 39–65% experience no change in migraine frequency under hormonal treatment [19]. A worsening of migraine appears to be more often with the use of COC in the classical 21-7 cycle, whereas progestogen-only treatment schemes, as well as COC used in the long-term cycle, are associated with an improvement in migraine burden [17,20,21,22]. As part of the treatment of a migraine patient with oral contraceptives, an adjustment of the hormonal strategy may be necessary over time. Accordingly, almost all participating gynecologists answered to have already changed and/or discontinued HC due to migraine. Interestingly, more than 3/4 of participants stated to have already started HC due to migraine and thus were actively involved in the prophylactic migraine treatment. However, for further migraine treatment the majority of gynecologists would refer to a neurologist. This highlights the close overlap between the specialties of neurology and gynecology with regard to the patient collective of young women suffering from migraine. The reality of everyday clinical practice indicates that one often works past each other. In a survey among 115 women’s healthcare providers in Connecticut, only 6% reported to be aware of migraine treatment guidelines and only 37% ever received headache-specific education [23]. A closer cooperation between neurologists and gynecologists would certainly be in the patients’ best interests and should definitely be strived for.

Despite comparable response rates in other surveys within the German outpatients setting [24,25], a limitation of the present study is the overall low response rate of 7.2%. Considering this low response rate, our results must be interpreted with caution when transferred to the entirety of practicing gynecologists in Germany. Potential motives for the low participation rate might include: 1. Lack of approachability—many of the contact addresses used were functional e-mail addresses, which in some cases are only rudimentarily read. In addition, a referral to the physician could not be guaranteed, even if this was actively requested in the cover letter; 2. Lack of time capacities—gynecologists having up to 150 patients per week and, correspondingly, up to 30 patients per day implies a lack of time for additional commitments, such as participation in a survey; 3. Request from non-gynecologists may be read with less interest; 4. Lack of scientific interest in migraine. Taking these factors into account, the population described in this study likely consists of gynecologists with a generally high interest in research and/or those with a particular interest in the topic of hormonal treatment for migraine, which could have led to a selection bias. In addition, the gender ratio of practicing gynecologists in Germany is approximately 1/3 men and 2/3 women. In our study cohort, the ratio is somewhat more pronounced, with 20% to 80% women. Possibly, female gynecologists are more willing to participate in a questionnaire study and may have a greater interest in migraine due to the higher prevalence of migraine in women.

Moreover, the design of an observational study based on a self-report questionnaire and the nature of the questions asked may have led to responses that are socially desired. However, anonymity should have reduced social desirability bias. In addition, participants were asked to answer truthfully and not to modify their answers to the recommendations of the current guidelines. Finally, it should be noted that the questionnaire was newly developed and not validated.

To overcome the obstacle of low response rates, future studies could investigate the actual intake of hormonal contraception in female patients with migraine of childbearing age. Bypassing the obstacle of practitioner’s feedback, this approach might yield more representative, albeit indirect, insight into HC prescribing behavior of gynecologists in patients suffering migraine. Yet such an approach would lack valuable information on the motives of drug selection, which were included in the presented study. Therefore, it should complement rather than replace questionnaire-based studies.

## 5. Conclusions

Our findings show that German gynecologists who responded to our questionnaire actively consider migraine before and while prescribing hormonal contraceptives, and that the diagnosis of migraine influences their prescribing behavior. Although PM is not associated with additional stroke risk, investigated gynecologists remained reluctant to prescribe this estrogen-free contraception for migraine with aura. Future studies could show whether prescription behavior differs in neurologists treating migraine in women taking hormonal contraception. Ultimately, improved interdisciplinary collaboration between gynecologists and neurologists might improve migraine treatment in in young female patients suffering migraine of childbearing age.

## Figures and Tables

**Table 1:** Demographic characteristics.

Age in Years, Mean ± SD (Min–Max; *n*)	52 ± 8 (33–78; 775)
Sex, *n* (%)	845
Male Female Divers	175 (21) 669 (79) 1 (0.1)
Work location, *n* (%) Rural Small town (<100,000 residents) City (100,000–500,000 residents) Large city (>500,000 residents)	846 170 (20) 317 (37) 185 (22) 174 (21)
Work experience in years, *n* (%) <5 5–10 11–20 >20	847 144 (17) 161 (19) 276 (33) 266 (31)
Clinical focus, *n* (MC) General gynecology, *n* (% of cases) Oncology, *n* (% of cases) Endocrinology, *n* (% of cases) Reproductive medicine, *n* (% of cases) Other focus, *n* (% of cases)	842 811 (96) 34 (4) 37 (4) 14 (2) 31 (4)
Weekly number of patients, *n* (%) (MC) <50 50–100 101–150 151–200 >200	844 41 (5) 256 (30) 338 (40) 149 (18) 60 (7)

SD = standard deviation of the means, MC = multiple choice allowed.

**Table 2:** Factors limiting HC prescription in concomitant migraine.

Influencing Factors	HC with COC % (*n*/Total *)	HC with PM % (*n*/Total *)
Cardiovascular risk	78 (494/633)	66 (97/147)
Migraine severity	60 (373/633)	53 (78/147)
Migraine frequency	61 (383/633)	50 (73/147)
Other comorbidities	71 (447/633)	N/A
Migraine treatment	26 (163/633)	29 (43/147)
others	23 (143/633)	39 (57/147)

* total = subgroup of gynecologists responding “depends on” when asking for prescription of hormonal contraception in migraine. N/A—not available.

**Table 3:** Factors limiting HC prescription in concomitant migraine with aura.

Influencing Factors	HC with COC % (*n*/Total *)	HC with PM % (*n*/Total *)
Cardiovascular risk	57 (36/63)	68 (224/332)
Migraine severity	57 (36/63)	51 (69/332)
Migraine frequency	56 (35/63)	49 (161/332)
Migraine aura severity	46 (29/63)	42 (140/332)
Migraine aura frequency	46 (29/63)	44 (147/332)
Other comorbidities	54 (34/63)	N/A
Migraine treatment	41 (26/63)	39 (129/332)
Others	38 (24/63)	28 (93/332)

* total = subgroup of gynecologists responding “depends on” when asking for prescription of hormonal contraception in migraineaura. N/A—not available.

## Data Availability

The datasets used and/or analyzed during the current study are available from the corresponding author on reasonable request.

## Supplementary Materials

The following supporting information can be downloaded at: https://www.mdpi.com/article/10.3390/jcm12041434/s1, File S1—questionnaire.
