# Supplementary material for: Migraine and Hormonal Contraception in Gynecological Outpatient Care—Cross-Sectional Study among Practicing Gynecologists in Germany
Source: J Clin Med. 2023 Feb 10;12(4):1434. doi: 10.3390/jcm12041434 (PMC9958685; doi:10.3390/jcm12041434)
Supplement: Supplementary file 1 [file jcm-12-01434-s001.zip › jcm-2208741-supplementary.pdf]

Supplementary Materials—File S1—questionnaire

Translated from German

Study information

Welcome to our study!

Thank you for your interest in our study "Migraine and hormonal contraception in gynecological practice."

By means of an anonymous survey, we would like to investigate the extent to which the presence of a migraine influences the decision to prescribe hormonal contraception. Answering all questions will take an average of 10 min (± 5 min, depending on the speed of answering). Please note that there is no "back button" when answering the questions. We are interested in clinical reality. When answering the questions, please refer to your daily medical practice.

Detailed information about the study and data protection according to EU-DSGVO can be found below. The survey is completely voluntary and can be cancelled at any time.

Please scroll all the way down to consent to data use and begin the survey.

Have fun filling it out! Your study team

[Attachment: "Study Information.pdf"]

Voluntariness of participation

Participation in this study is voluntary. You may discontinue the study at any time, even without giving reasons. You can withdraw your consent at any time, also without giving reasons. You will not suffer any disadvantages as a result.

[Attachment: "Study Information.pdf"]

I agree that my personal data will be processed according to the information given here

YES

NO

(Only if you agree, you can participate in our survey. If you do not want to agree, you can close the window now.)

Informed consent

Declaration of consent for participation in the study "Migraine and hormonal contraception in gynecological practice."

I hereby declare that I have been informed in written form about the nature, significance, and risks of the scientific investigations in the study and have had sufficient opportunity to clarify potential questions with the study team. I understand that I have the right to withdraw my consent at any time without giving reasons and without adverse consequences for me, and to object to further processing of my data and request its destruction.

You can download the consent statement below as a PDF.

Please agree to the following three statements to begin the study: [Attachment: “Informed Consent.pdf”]

I hereby confirm that:

I am at least 18 years old

No, I am a minor

I also confirm that:

I am voluntarily willing to participate in the scientific study.

No, I do not wish to participate.

I agree that:

The results of the study may be published in an anonymous form that does not allow any conclusions to be drawn about my person, will be published.

No, I do not agree.

General information (page 1/5)

We will start with some general questions about your job.

What is your gender? (male, female, diverse)

1. How old are you (in years)? ________________________________
2. In what state do you work?
    - o Baden-Wuerttemberg
    - o Bavaria
    - o Berlin
    - o Brandenburg
    - o Bremen
    - o Hamburg
    - o Hessen
    - o Mecklenburg-Western Pomerania
    - o Lower Saxony
    - o North Rhine-Westphalia
    - o Rhineland-Palatinate
    - o Saarland
    - o Saxony
    - o Saxony-Anhalt
    - o Schleswig-Holstein
    - o Thuringia
3. Where is your practice/primary work location?

- Rural area
- Small town (up to 100,000 inhabitants)
- Large town (100,000 - 500,000 inhabitants)
- Large town (over 500,000 inhabitants)

4. How long have you been working in gynecological outpatient care?
   - Less than 5 years
   - 5-10 years
   - 10-20 years
   - >20 years
5. How many patients do you see in an average week?
   - Fewer than 50
   - 50-100
   - 100-150
   - 150-200
   - >200
6. In what area of gynecology do you primarily work?
   - General gynecology
   - Gynecologic oncology
   - Gynecologic endocrinology
   - Reproductive medicine
   - Other and namely ___________________________________
7. How often do you prescribe hormonal contraception on work days (new and follow-up prescriptions)?
   - Several times a day
   - About once a day
   - Several times a week but less than once a day
   - Approximately once a week
   - Several times a month but less than once a week
   - About once a month
   - Less than once a month
   - Never

(You have indicated that you never prescribe hormonal contraception. Therefore, you may not participate in the rest of the survey. After clicking the submit button, the survey will be closed. Thank you for your interest!)

Migraine and combined contraception (page 2/5)

This page is about combined hormonal preparations (i.e., estrogen-progestin preparations). Please answer the questions as you would in your everyday medical practice.

1. Before you prescribe combined contraception ...

... how often do you actively ask about the presence of migraine?

- Always
- Often
- Sometimes
- Rarely
- Never

... how often do you specifically ask about the presence of migraine aura?

- Always
- Often
- Sometimes
- Rarely
- Never

2. Would you prescribe combined contraception for a patient with migraine without aura?
    - Yes, without restrictions
    - No
    - Depends on
3. What does that depend on? - multiple answers possible
    - Frequency of migraine
    - Severity of migraine
    - Therapy of migraine
    - Presence of cardiovascular diseases/risk factors
    - Concomitant diseases
    - Other
4. Please briefly explain your choice. (If you do not wish to provide further information, please enter x). ____________________

5. Would you prescribe combined contraception for a patient with migraine with aura?
    - Yes, without restrictions
    - No
    - Depends on
6. What does this depend on? - multiple answers possible

- o Frequency of migraine
- o Severity of migraine
- o Frequency of aura
- o Severity of aura
- o Treatment of migraine
- o Presence of cardiovascular disease/risk factors
- o Concomitant diseases
- o Other

7. Please briefly explain your choice. (If you do not want to give any further information, please enter x) ________________________________

Migraine and progestin monopreparations (page 3/5)

This page is about progestin-only preparations. Please answer the questions as you would in your everyday medical practice.

1. Before you prescribe a progestogen-only preparation ...

... how often do you actively ask about the presence of migraine?

- o Always
- o Often
- o Sometimes
- o Rarely
- o Never

... how often do you specifically ask about the presence of migraine aura?

- o Always
- o Often
- o Sometimes
- o Rarely
- o Never

2. Would you prescribe a progestogen monopreparation for a patient with migraine without aura?
   - o Yes, without restrictions
   - o No
   - o Depends on
3. What does that depend on? - multiple answers possible
   - o Frequency of migraine
   - o Severity of migraine
   - o Therapy of migraine
   - o Presence of cardiovascular disease/risk factors

- Others

4. Please briefly explain your choice. (If you do not wish to provide further information, please enter x). ___________________________________

5. Would you prescribe a progestogen monopreparation for a patient with migraine with aura?
   - Yes, without restrictions
   - No
   - Depends on

6. What does this depend on? - multiple answers possible
   - Frequency of migraine
   - Severity of migraine
   - Frequency of aura
   - Severity of aura
   - Treatment of migraine
   - Presence of cardiovascular disease/risk factors
   - Others

7. Please briefly explain your choice. (If you do not want to give any further information, please enter x) ___________________________________

Treatment decisions (page 4/5).

This is the second-to-last page. This is about therapeutic decisions related to hormonal contraception for migraine.

1. Have you ever started hormonal therapy for migraine?
   - Yes
   - No

2. Which therapy? - multiple answers possible
   - Treatment with combined contraception in the classic 21-7 cycle
   - Treatment with combined contraception in long-term cycle
   - Treatment with progestogen monotherapy other

3. Have you ever changed an existing hormonal therapy due to a migraine?
   - Yes
   - No

4. How? - multiple answers possible
   - o Combined hormonal therapy: from classic 21-7 cycle to long-term cycle
   - o Combined hormonal therapy: from long-term cycle to classic 21-7 cycle
   - o From combined hormonal therapy to progestogen monotherapy
   - o From progestogen monotherapy to combined hormonal therapy
   - o Different

5. Have you ever stopped hormonal therapy because of migraine?
   - o Yes
   - o No

6. Which therapy?
   - o Combined hormonal therapy in the classic 21-7 cycle
   - o Combined hormonal therapy in long-term cycle Progestogen monotherapy
   - o Other and namely: ____________________________________

7. Why exactly? - multiple answers possible
   - o Contraindicated for migraine
   - o Worsening of migraine during therapy
   - o Other reasons and namely: ____________________________________

Last questions (page 5/5)

And finally, two questions:

1. What do you do when a patient reports migraine?
   - o I treat the migraine myself.
   - o I make a referral to the primary care physician. I recommend seeing a neurologist. I don't do anything special.

2. Would you like more information/educational opportunities about migraine?
   - o Yes, definitely.
   - o Yes, but it will be difficult in terms of time.
   - o No, not interested.
   - o No, no time.
